# High prevalence of asymptomatic HBV chronic carriage in HIV infected long term survivors

**DOI:** 10.1186/1471-2334-14-S4-O16

**Published:** 2014-05-29

**Authors:** Aura Temereanca, Luminița Ene, Adelina Rosca, Camelia Grancea, Claudia Dita, Dan Duiculescu, Cristian L Achim, Simona Ruță

**Affiliations:** 1 Carol Davila University of Medicine and Pharmacy, Bucharest, Romania; 2 Ștefan S. Nicolau Institute of Virology, Bucharest, Romania; 3 Clinical Hospital of Infectious and Tropical Diseases “Dr. Victor Babeş”, Bucharest, Romania; 4 University of California at San Diego, La Jolla, California, USA

## 

Hepatitis B virus (HBV) infection is common in individuals infected with human immunodeficiency virus, and coinfection is associated with higher rates of HBV replication and more rapid liver disease progression than HBV monoinfection. This study evaluates the prevalence and virological profiles of hepatitis B infection in a cohort of long term survivors, with multiple antiretroviral treatments.

164 HIV-infected subjects (median age: 24 years) on combined antiretroviral therapy (cART) (median duration: 13 years), were evaluated for serologic markers of HBV infection (HBsAg, total anti-HBc and anti-HBsAg antibodies). Markers of HBV infectivity (HBeAg and HBV DNA) were evaluated in all HBsAg carriers; HBV genotype and lamivudine resistance mutations were analyzed in the cases with HBV DNA >10^3^ IU/mL.

65.9% of the patients (108/164) had markers of past or present HBV infection (antiHBc positives), out of which 51.8% (56/108) were chronic HBV carriers and 30.5% had resolved HBV infection. All subjects were equally exposed to HBV infection, irrespective of their current immune status. Out of 21 patients with isolated anti-HBc antibodies, only 4 had detectable HBV DNA, presumably having occult hepatitis B. HBV chronic carriage rate was not influenced by the immune status. Overall, only 17.8% of the chronic carriers had active HBV replication; severely immune-depressed patients tend to maintain active viral replication more frequently than those with moderate or absent immunosuppression. The majority of the coinfected individuals (68.3%) showed no sign of liver fibrosis (APRI score <0.5), only 3% had severe fibrosis (APRI score >1.5); HBV DNA was directly correlated with APRI score. HBV genotype A was present in all but one of the tested patients. 98.8% of the coinfected subjects have been treated with a cART regimen that includes a drug dually active against HIV and HBV (in 98% of the cases lamivudine (3TC), for a mean time of 6.9 years and in 29.7% of the cases the current dually active drug was tenofovir). 3TC-resistance mutations were present in only 4 coinfected subjects.

We found a strikingly low percentage of long term HIV/HBV coinfected patients from our group with active liver disease. A high prevalence of asymptomatic HBV chronic carriage was associated with a good immune status, suggesting that dually active antiretrovirals have an important role in delaying progression of liver disease in HIV/HBV coinfected patients.

